# Effectiveness of an antibacterial primer used with adhesive-coated brackets on enamel demineralization around brackets: an in vivo study

**DOI:** 10.1186/s40510-019-0271-3

**Published:** 2019-04-15

**Authors:** Aslihan Zeynep Oz, Abdullah Alper Oz, Sabahat Yazicioglu, Ozlem Sancaktar

**Affiliations:** 1 Department of Orthodontics, Faculty of Dentistry, Ondokuz Mayıs University, 55139 Samsun, Atakum Turkey; 2 Dental Care Hospital, Samsun, Turkey

**Keywords:** Antibacterial primer, White spot lesions, Oral hygiene

## Abstract

**Background:**

The aim of the study is to assess the clinical effect of an antibacterial monomer-containing primer on preventing white spot lesions (WSLs) during fixed orthodontic treatment.

**Subject and methods:**

The study included 35 patients. A split-mouth design was used during bonding of the brackets. In Clearfil (CF) group, adhesive-coated brackets (APC Plus Victory series, 3M Unitek, Monrovia, CA, USA) were bonded with an antibacterial monomer-containing primer (Clearfil Protect Bond, Kuraray Medical, Okayama, Japan). In Transbond (TB) group, the same adhesive-coated brackets were bonded using a conventional primer (Transbond XT Primer; 3M Unitek, Monrovia, CA, USA). The mean duration of orthodontic treatment was 16 months. Digital images of each tooth were used to assess the WSLs. The areas of the WSLs were measured with a software. The bond failures during orthodontic treatment were also recorded.

**Results:**

After fixed orthodontic treatment, 23 of the 35 patients showed one or more WSLs. Of the total of 666 teeth, 114 WSLs occurred over the orthodontic treatment time. Rates of WSL in the CF and TB groups were 8.03% and 9.24%, respectively. The difference in WSL rates between the two groups was not statistically significant. No significant difference was observed in the lesion areas between the groups. Moreover, the difference in bracket failure rates between the two groups was also not statistically significant.

**Conclusion:**

The results of this long-term clinical study indicated no significant difference between the antibacterial monomer-containing primer group and the control group in the efficacy of reducing demineralization throughout the orthodontic treatment.

## Introduction

White spot lesions (WSLs) are the first sign of demineralization of the enamel surface of a tooth, and preventing them during fixed appliance therapy is a challenge for the orthodontist. Fixed orthodontic appliances have irregular surfaces that make tooth cleaning more difficult and create areas favorable to plaque accumulation [1]. Prolonged plaque accumulation causes WSLs to develop, and their prevalence in orthodontic patients ranges from 2 to 96% after fixed appliance treatment [2–6].

Patient education is the first step in preventing formation of WSLs. Professional oral hygiene training and regular professional cleaning have been reported to be effective in reducing decalcification in patients who have low levels of compliance [7]. During orthodontic treatment, fluoride may be administered through toothpaste, mouth rinses, and gels [8]. Application of topical fluoride and casein phosphopeptide-stabilized amorphous calcium phosphate (CPP-ACP) nanocomplexes can help to heal or remineralize small lesions [9].

Resin-filled sealants, fluoride-releasing adhesives, and antibacterial adhesives may decrease the occurrence of WSLs without patient compliance [10, 11]. In addition, nanoparticles (nanofillers, silver, TiO_2_, SiO_2_, hydroxyapatite, fluorapatite, fluorahydroxyapatite) can be used to prevent microbial adhesion or enamel demineralization around orthodontic brackets [12]. However, the literature includes a limited number of studies of the long-term clinical effectiveness of these adhesives. Most such studies investigated these products after the brackets had been in the oral cavity for weeks or months [11]. As we know, fluoride-containing resins do not show the same fluoride-releasing capacity 90 days after first application [13]. For this reason, these resins should be applied repeatedly to increase their effectiveness [14, 15]. If a certain material can absorb fluoride (e.g., glass ionomer), fluoride toothpaste can supply the fluoride repetitively. However, there is no certain evidence regarding the duration of the effect on WSLs of an antibacterial monomer-containing primer. One study showed that using an antibacterial monomer-containing primer to bond orthodontic attachments inhibited caries [13]. However, the investigation period in that study was only 30 days. Thus, the long-term results are uncertain.

Long-term clinical studies could provide more relevant data on decreasing WSL formation around brackets and might be an appropriate guide for clinical practice. Therefore, this clinical study aimed to investigate the long-term efficacy of an antibacterial monomer-containing primer when used with an adhesive-coated bracket system to prevent demineralization adjacent to bracket margins and compare this primer with a conventional one when used with the same brackets during orthodontic treatment, and record and compare the clinical bond failure rates of the brackets. The null hypothesis was that there was no difference in the occurrence of WSLs on enamel surfaces whether they were bonded with antibacterial monomer-containing primer or conventional primer.

## Subject and methods

This study was approved by the regional ethics committee (OMUKAEK 2016/193). The study included 35 patients with a mean age of 14.4 years. They were selected according to the following criteria: (1) no visible WSL on the buccal enamel surfaces of the teeth, (2) permanent dentition, (3) no restoration of the buccal surfaces of the teeth, and (4) good oral hygiene at the beginning of the fixed orthodontic treatment. The study did not include the teeth that were extracted according to the patient’s orthodontic treatment plan.

All teeth were cleaned and polished before the fixed appliances were bonded. In the Clearfil (CF) group, teeth were acid etched with 32% phosphoric acid (Scotchbond™ Universal Etchant, 3M Dental Products, Germany) for 10 s, rinsed, and dried. Then an antibacterial and fluoride-releasing self-etching primer (Clearfil Protect Bond, Kuraray Medical, Okayama, Japan) was used according to the manufacturer’s instructions, and adhesive-coated brackets (APC Plus Victory series, 3M Unitek, Monrovia, CA, USA) were bonded. Clearfil Protect Bond has two properties: long-term fluoride release and 12-methacryloyloxydodecylpyridinium bromide (MDPB), which has an antibacterial effect. Although Clearfil is a self-etching primer, the manufacturer suggests applying 35% phosphoric acid to the uncut enamel and letting it remain for 10 s, before washing and drying. In the Transbond (TB) group, the teeth were acid etched with 32% phosphoric acid for 30 s, rinsed, and dried. Then, a conventional primer (Transbond XT Primer; 3M Unitek, Monrovia, CA, USA) was applied to the etched enamel surfaces, and the same adhesive-coated brackets were bonded. The Transbond XT primer has no fluoride-releasing property. The adhesive remaining around the bracket margins was removed. All bonding procedures were performed by the same investigator (A.A.O) who was blind to which material was used on which side during and after the procedures.

Another investigator (O.S.) provided all patients with oral hygiene instructions and brushing training. The patients were also prescribed with fluoride toothpaste. After the fixed orthodontic appliances were removed, the adhesive remaining on the enamel surfaces was removed using a carbide-finishing bur. The mean duration of orthodontic treatment was 16 months.

**Fig. 1:**
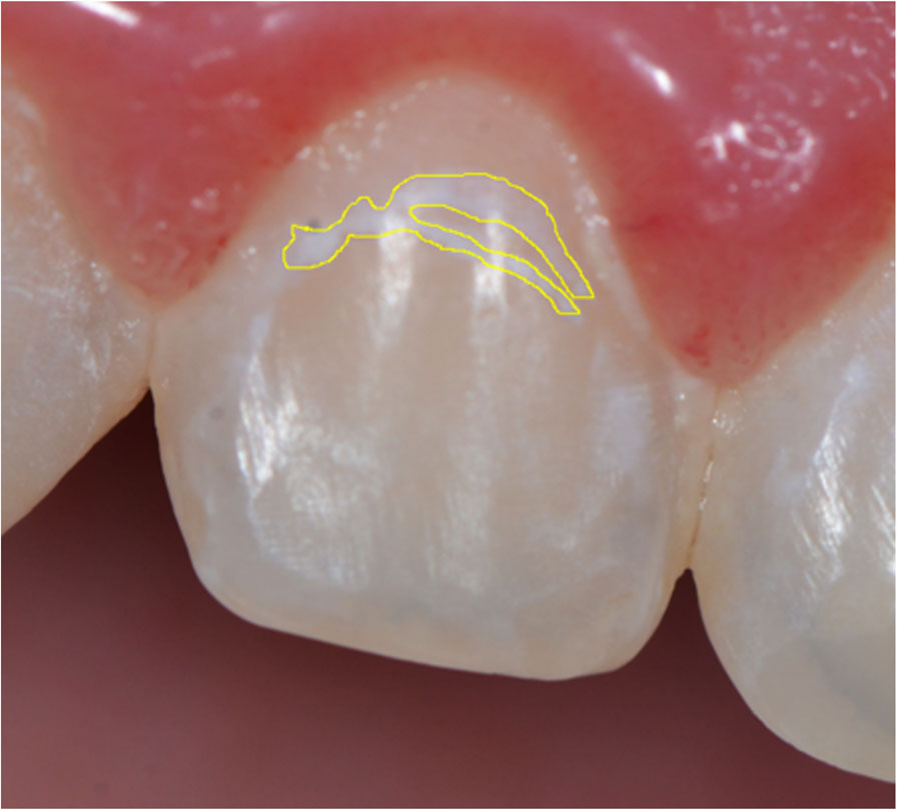
The areas of WSLs were calculated with a software

Standard clinical photographs were taken before and after orthodontic treatment according to the American Board of Orthodontics [16]. In addition, photos taken of each tooth individually before and after the orthodontic treatment showed the buccal surfaces of the premolars, canines, and all incisors. The same examiner (A.A.O) used those individual photographs to examine all WSLs presence both before and after orthodontic treatment. Patients with enamel hypoplasia, demineralization before the treatment, and other developmental alterations were excluded from the study. All examinations were conducted using the open-source image processing software (Image J, version 2.0, National Institutes of Health, Bethesda, MD, USA). The images that showed WSLs were scaled according to the mesiodistal measurements of each tooth as measured on an orthodontic model (Fig. 1). Then the actual areas of the WSLs were measured using the same software. The reliability of these measurements was assessed by having the same investigator recalculate the measurements of 10 patients selected at random. The mean error was 0.11 mm^2^ for the lesion areas.

The presence and severity of the WSLs were also recorded by visually assessing the photographs. The lesions were scored as follows: 0 = no WSLs, 1 = slight WSLs, 2 = severe WSLs covering more than one third of the surface, and 3 = WSLs with cavitation.

The clinical failure rates were also recorded during the fixed therapy. Each patient was recalled every month (4–5 weeks) for a routine appointment. First-time bracket failures were recorded. If a bracket broke, a new one was bonded at the first appointment after the failure. New brackets were bonded using the bonding protocols used at the beginning of the treatment for that patient’s investigation group. However, new brackets were not included in the bond-failure section of the study.

A split-mouth design was used to bond the brackets, allowing each patient to be his or her own control. Each patient’s oral cavity was divided into four quadrants. In 18 randomly selected patients, the teeth on the maxillary right and mandibular left quadrants were used as control sides (which received conventional primer), and the maxillary left and mandibular right quadrants of the dental arches were used as treatment sides (which received antibacterial and fluoride-releasing self-etching primer). For bonding the other 17 patients’ brackets, the quadrants were inverted. To eliminate any bias, the sides bonded with Clearfil Protect primer (CF group) and those bonded with Transbond XT primer (TB group) were alternated on each consecutive patient.

### Statistical analysis

Statistical analyses were performed using a software package (IBM SPSS version 23, Chicago, IL, USA). Wilcoxon’s test was used to compare the areas of the WSLs between the groups. Bracket survival rates during orthodontic treatment were evaluated with the Kaplan–Meier test. Differences in bracket survival curves by primer type, tooth type, dental arch, and patients’ sex were evaluated with the log-rank test. Chi-square tests were used to analyze the relationship between frequencies of WSLs and primer type. The level of significance was set at *P* < 0.05.

## Results

Table 1 shows the characteristics of the sample and the distributions of the bracket types, primers, and sexes. After the fixed orthodontic treatment, 23 of the 35 patients showed one or more WSLs. In all, 34 premolar teeth were extracted according to the patients’ orthodontic treatment plan. On the 666 total teeth, 114 WSLs occurred over the course of the full orthodontic treatment time. The incidence of WSLs for the CF and TB groups were 8.03% and 9.24%, respectively. The difference in WSL incidence between the two groups was not statistically significant. Table 2 shows the distribution of the WSL incidence according to the groups and the tooth type. In both groups, more WSLs were seen in the lateral incisors.

**Table 1:** Identification of the samples

	Number	Percentage (%)
Patients		
Female	12	38.3
Male	23	61.2
Primer type		
CF	333	50
TB	333	50
Location		
Maxillary brackets	315	47.3
Mandibular brackets	317	52.7
Tooth type		
Maxillary incisor	140	21
Mandibular incisor	140	21
Maxillary canine	70	10.5
Mandibular canine	70	10.5
Maxillary premolar	122	18.3
Mandibular premolar	124	18.6

*CF* Clearfil, *TB* Transbond

**Table 2:** Distributions of the WSLs according to tooth type

		CF	TB
Maxilla	Second premolar	3	4
	First premolar	5	5
	Canine	6	9
	Lateral	15	15
	Central	6	10
Mandibular	Second premolar	5	7
	First premolar	6	6
	Canine	5	4
	Lateral	1	1
	Central	1	–
Total		53	61

*CF* Clearfil, *TB* Transbond

### Area of WSLs

In the upper arch, the mean WSL area was 2.24 mm^2^ in the CF group and 2.72 mm^2^ in TB group; in the lower arch, it was 1.78 mm^2^ in the CF group and 2.22 mm^2^ in the TB group (Table 3). No significant differences between the two groups were observed in either the WSL area or the rates of bracket failure. In addition, there was no significant difference between the groups in the WSL score frequency (Table 4).

**Table 3:** Comparison of the white spot lesion area (mm^2^)

	Group	Number of WLS	Mean	SD	Min	Max	*P*
Maxilla	CF	35	2.24	1.81	0.28	7.91	0.310
	TB	43	2.72	2.18	1.18	10.85	
Mandibular	CF	18	1.58	1.76	0.21	7.14	0.170
	TB	18	2.22	2.09	0.11	8.79	
Total	CF	53	2.01	1.80	0.21	7.91	0.051
	TB	61	2.57	2.16	0.12	10.85	

*CF* Clearfil, *TB* Transbond

*P* > 0.05

**Table 4:** Comparison of frequency of WSLs scores according to the groups

	0	1	2	3	*P*
CF group	272 (83.7)	39 (12.0)	13 (4.0)	1 (0.3)	0.820
TB group	280 (82.1)	41 (12.0)	18 (5.3)	2 (0.6)	
Total	552 (82.9)	80 (12.0)	31 (4.7)	3 (0.5)	

*CF* Clearfil, *TB* Transbond

*P* < 0.05

### Bracket failure

No significant difference was observed among the dental arches and sex in the bracket failure rates. However, the rates of bond failure were higher for premolars (6.09%) than for incisors (1.07%) and canines (1.42%) (Table 5).

**Table 5:** Distribution of the bracket failure rates

	Number	Bracket failure	Censored	Percent of censored	Failure rate (%)	Log-rank
Adhesive type						
Clearfil	333	11	323	96.7	3.30	0.316
Transbond	333	9	323	97.3	2.70	
Dental arch						
Maxillary	332	8	324	97.6	2.54	0.622
Mandibular	334	12	322	96.4	3.79	
Bracket type						
Incisor	180	3	277	98.9	1.07	0.046^*^
Canine	140	2	138	98.6	1.42	
Premolar	246	15	231	93.9	6.09	
Sex						
Female	446	10	428	97.7	2.24	0.303
Male	220	10	218	95.6	4.55	

*Indicates statistically significant (*P* < 0.05)

## Discussion

Microbial dental plaque potentially increases the incidence of WSLs. Excess adhesive around the bracket is another factor facilitating plaque accumulation. Therefore, removal of the adhesive around the bracket may reduce the amount of plaque accumulation and thus reduce the WSLs [17, 18]. For this purpose, adhesive-coated appliance systems with uniform-coating adhesive on each bracket have become available. These products allow easy flash clean up. One goal of such products is to achieve a sufficient marginal seal using less bonding adhesive around the bracket margins. In the present study, adhesive-coated systems were used in both groups to eliminate the effect of adhesive around the bracket.

The composite used with these adhesive-coated brackets is a polyacid-modified composite resin that releases a small amount of fluoride to enhance patient confidence. However, there is no information regarding how long the fluoride release continuous. The present study used these brackets in all quadrants in all patients. Clearfil bond has fluoride-releasing properties and also includes MDPB, which has an antibacterial effect. In contrast, the conventional primer, Transbond XT, has no fluoride-releasing property. The split-mouth design may eliminate the factors that differ among individuals including diet, oral hygiene, and saliva pH.

Uysal et al. used the same antibacterial monomer-containing primer to bond orthodontic brackets and indicated that this primer inhibited caries in vivo [11]. However, the investigation period was 30 days and this short period did not successfully simulate the full orthodontic treatment period. In the present study, the mean duration of the orthodontic treatment was 16 months; thus it is inappropriate to compare the results of the present study with those of Uysal et al. The present study’s results indicate that the combination of antibacterial primer and adhesive-coated brackets that released an amount of fluoride had no significant effect to prevent enamel demineralization during orthodontic treatment when compared with the combination of conventional primer and adhesive-coated brackets. It is possible that the split-mouth design of the present study may affect the results because of a release of fluoride from materials that were used. Although Benson [19] and Lesaffre et al. [20] emphasized the possibility of cross-contamination between sides, another study found that applied topical fluoride application exerted most of its effect locally [21]. This difference may also be due to the small sample size. Although it used a split-mouth design, the study included only 35 patients. In addition, oral hygiene status may be the most effective factor in preventing WSLs. The patients in the present study had good oral hygiene at the baseline, and they maintained it at an acceptable level throughout the orthodontic treatment. The results might differ in the case of patients who have very poor oral hygiene.

Another recent study also investigated the same antibacterial adhesive and compared its efficacy with that of another fluoride-recharging adhesive and that of a conventional orthodontic adhesive. That study used the micro-computed tomography (micro-CT) method and an investigation period of 8 weeks. It found no significant differences among the adhesives regarding either the volume or depth of the WSLs. The results of the present study, which was long-term, were similar results to those of this short-term in vivo study [22].

Studies in the literature report differing levels of the prevalence of WSLs. Their results may have been affected by the difference in the materials, length of the evaluation period, investigation method, and area evaluated. Lovrov et al., which like the present study, also used photographs and reported a 26% prevalence [23]. In the present study, the WSL rates for the CF and TB groups were 8.03% and 9.24%, respectively. Although there was no significant difference between the groups, both showed WSL rates much lower than those in the study by Lovrov et al. In another study, the prevalence was higher, 36%, but only maxillary anterior teeth were evaluated [24]. In the present study, the maxillary teeth showed more WSLs than the mandibular teeth, a result that indicates that maxillary teeth are more likely to have a greater number of enamel lesions after orthodontic treatment [3]. Most previous studies have shown that maxillary laterals or canines are the teeth most commonly affected [3, 23], and the present study found similar results.

The most common index used to determine the presence, absence, or severity of WSLs was a 0–3 scoring system [25, 26]. In the literature, most studies of WSLs used two different assessment methods: intraoral photography and visual assessment [5]. Photographs are advantageous for assessing of WSLs because different researchers can evaluate the images under magnification, making it is possible to see small WSLs that may be overlooked during visual evaluation. However, the light used to take the photograph may mask the WSLs. In the present study, we tried to take standard image records using the same camera and equipment. The main advantage of the photographs was to enable measurement of the WSL areas. Because the scoring system mentioned above cannot effectively address lesion size, we measured the areas of the lesions to determine whether one group had an advantage in reducing the WSLs. The teeth identified as having WSLs were analyzed in terms of WSL area using Image J software, which was also used in a previous study [24].

The rate of bracket failure is another important factor for clinicians when deciding which materials to use. The literature describes some bonding materials that reduce the incidence of enamel decalcification around the brackets, but their failure rate was higher than in the control groups and their bonding performances may not be acceptable for clinicians [27–29]. In the present study, the failure rate was 2.70% for the CF group and 3.30% for the TB group; these failure rates were not high compared to those of past studies [25]. The present study also evaluated the effects of bracket location, bracket type, and patient sex on failure rate. The results showed that only bracket type had a significant effect on the rate of bracket failure. The premolars showed the highest failure rate (6.09%). The risk of moisture contamination and occlusal forces are higher in posterior teeth than in anterior ones; thus, the failure rate of posterior brackets is usually higher than anterior brackets [30, 31].

The present study showed that using an antibacterial monomer-containing primer with adhesive-coated brackets has no significant advantage in reducing enamel demineralization when compared with a combination of conventional primer and adhesive-coated brackets over the duration of the full orthodontic treatment. Nevertheless, further studies with larger sample sizes should be conducted to identify the most promising materials to include in studies of the clinical effects of fluoride-releasing and antibacterial materials on the prevention of WSLs.

## Conclusion

- There is no significant difference between the group using antibacterial monomer-containing primer group and the control group regarding efficacy in reducing demineralization over the full course of orthodontic treatment.
- The most effective method for preventing WSLs during orthodontic treatment is still considered to be good oral hygiene.

## Abbreviations

CF: Clearfil
CPP-ACP: Casein phosphopeptide-stabilized amorphous calcium phosphate
MDPB: 12-Methacryloyloxydodecylpyridinium bromide
TB: Transbond
WSLs: White spot lesions
